# Bias Tunable Photocurrent in Metal-Insulator-Semiconductor Heterostructures with Photoresponse Enhanced by Carbon Nanotubes

**DOI:** 10.3390/nano9111598

**Published:** 2019-11-11

**Authors:** Antonio Di Bartolomeo, Filippo Giubileo, Alessandro Grillo, Giuseppe Luongo, Laura Iemmo, Francesca Urban, Luca Lozzi, Daniele Capista, Michele Nardone, Maurizio Passacantando

**Affiliations:** 1 Physics Department “E.R. Caianiello”, University of Salerno, via Giovanni Paolo II 132, 84084 Fisciano, Salerno, Italy; agrillo@unisa.it (A.G.); giluongo@unisa.it (G.L.); liemmo@unisa.it (L.I.); furban@unisa.it (F.U.); 2 CNR-SPIN Salerno, via Giovanni Paolo II 132, 84084 Fisciano, Italy; filippo.giubileo@spin.cnr.it; 3 Department of Physical and Chemical Science, University of L’Aquila, via Vetoio, 67100 Coppito, L’Aquila, Italy; luca.lozzi@aquila.infn.it (L.L.); daniele.capista@student.univaq.it (D.C.); michele.nardone@aquila.infn.it (M.N.); maurizio.passacantando@aquila.infn.it (M.P.)

**Keywords:** carbon nanotubes, heterostructures, photoconductivity, Schottky junctions, MISIM

## Abstract

Metal-insulator-semiconductor-insulator-metal (MISIM) heterostructures, with rectifying current-voltage characteristics and photosensitivity in the visible and near-infrared spectra, are fabricated and studied. It is shown that the photocurrent can be enhanced by adding a multi-walled carbon nanotube film in the contact region to achieve a responsivity higher than $100\;\mathrm{mA}\;W^{-1}$ under incandescent light of $0.1{\;\mathrm{mW}\;\mathrm{cm}}^{-2}$. The optoelectrical characteristics of the MISIM heterostructures are investigated at lower and higher biases and are explained by a band model based on two asymmetric back-to-back Schottky barriers. The forward current of the heterojunctions is due to majority-carrier injection over the lower barrier, while the reverse current exhibits two different conduction regimes corresponding to the diffusion of thermal/photo generated carriers and majority-carrier tunneling through the higher Schottky barrier. The two conduction regimes in reverse bias generate two plateaus, over which the photocurrent increases linearly with the light intensity that endows the detector with bias-controlled photocurrent.

## 1. Introduction

Carbon nanotubes (CNTs) have been attracting a lot of attention in the past three decades due to their remarkable chemical, mechanical, and electrical properties [1,2,3,4]. In both single-walled (SWCNT) and multi-walled (MWCNT) forms, carbon nanotubes have been considered in several electronic applications, such as transistors [5,6,7,8], diodes [9,10], memory devices [11,12], photovoltaic cells [13,14], photodetectors [15,16,17], strain [18] and chemical sensors [19,20,21,22], and field emitters [23,24,25,26], etc.

Although standalone CNT photodetectors have shown limited performance, low-noise and enhanced light detection can be achieved by combining nanotubes with traditional semiconductors in hybrid devices [27,28]. The CNT type, whether single- or multi-walled, their quality and configuration in bundles or films of different density and thickness as well as the role of the semiconductor substrate in such hybrid structures have been widely investigated [14,29,30,31]. In this context, photodetectors based on MWCNT [32,33,34] or SWCNT [35,36] films over Si have been the preferred devices for their fast response and high detection capability combined with easy fabrication, low cost, high reliability, and compatibility with existing technologies. Optimized devices realized with SWCNT films have achieved time response and photoresponsivity in the order of 10 μs and $1{\;\mathrm{AW}}^{-1}$ under LED light (460 nm) at 2 ${\mathrm{mW}\;\mathrm{cm}}^{-2}$ incident power [37]. 

CNT films in contact with Si form rectifying junctions where the CNTs, owing to their high electrical conductivity and optical transparency, work both as an antireflective layer and conductive electrode for photocharge collection [38,39,40,41]. Depending on the thickness, density, and orientation, the CNT film can also play a role in the photocharge generation and injection over the junction, especially under low-energy irradiation [42,43].

The CNT/Si photodetector fabrication includes the formation of metal contacts on the CNT film and the Si substrate. Care and expensive processing are required to make these contacts ohmic and low resistive [44]. Very often, the ohmic behavior is taken for granted as a needed simplification for the analysis of the electrical behavior. Only a few studies have considered that extra Schottky junctions can be formed by the contacts, and none have investigated such a possibility in depth [32,45]. 

With the present study, we try to fill this gap by studying a Pt-Ta_CNT/Si_3_N_4_/n-Si/Si_3_N_4_/Ta-Pt MISIM heterostructure, with the top Pt-Ta contact connected to a film of MWCNTs. We apply electrical stress to transform the two insulating layers in conductive barriers and study the optoelectrical response of the so-obtained device both in the low and high bias regimes. The structure, described by two back-to-back asymmetric Schottky barriers, behaves as a diode with forward current due to electrons injected over the lower barrier and reverse current exhibiting two conduction regimes attributed to thermal/photo generation and to electron tunneling over the junction with higher Schottky barrier. The two conduction regimes in reverse bias cause two plateaus and enable the control of the photocurrent gain by bias. The photocurrent increases linearly with the light intensity and is generated mainly in the n-Si substrate. The presence of a MWCNT film in the contact region enhances the photoresponse of the device, mainly by increasing the photosensitive area of the junction.

## 2. Materials and Methods 

**Figure 1:**
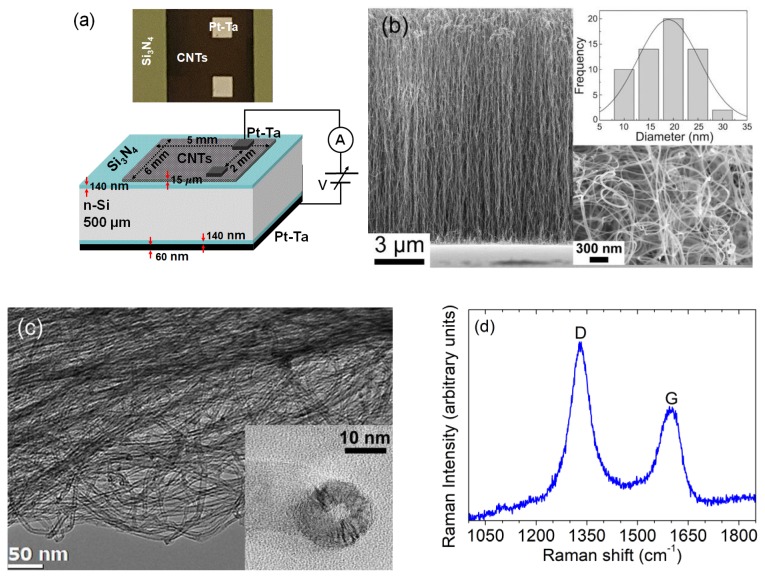
(**a**) Top image of the device after the multi-walled carbon nanotube (MWCNT) growth (black area) and layout of the device with the setup used for the electrical characterization of the Pt-Ta_CNT/Si_3_N_4_/Si/Si_3_N_4_/Pt-Ta heterostructure. (**b**) Scanning electron microscope (SEM) image of the MWCNT film. The insets show the distribution of the outer diameter (top inset) and the top view of the film (bottom inset). (**c**) Transmission electron microscope and high-resolution transmission electron microscope (HRTEM) (bottom inset) images of the MWCNTs. (**d**) Raman spectrum of the MWCNT film.

The layout of the device under study, as seen in Figure 1a, consists of an n-type Si substrate (resistivity 1–5 Ω cm, doping $∼10^{15}{\;\mathrm{cm}}^{-3}$) with the top and bottom surfaces covered by 140 nm thick Si_3_N_4_ layers. Two 1 ${\mathrm{mm}}^{2}\;$Pt-Ta pads (50 nm Pt over 10 nm Ta) are sputtered at a distance of 2 mm from each other on the top side and immersed in a $6\times 5{\;\mathrm{mm}}^{2}$ film of MWCNTs. A film of Pt-Ta covers the entire bottom side of the substrate.

The growth of the CNT film on a specific area of the Si substrate started with the thermal evaporation of a 3 nm thick Ni film under a pressure of $10^{-6}\;$Torr. Ni was used as the catalyst for the selective chemical vapour deposition (CVD) of the nanotubes [46]. The partially Ni-coated substrate was inserted in a quartz CVD reactor that was pumped down to less than 10^-7^ Torr using a turbo molecular pump. In order to form the catalytic particles in the nanometer size, the substrate was pre-treated in H_2_ gas with a flow rate of 5 sccm for 10 min at 750 °C. The MWCNTs were grown by adding C_2_H_2_ at a flow rate of 20 sccm for 20 min at the same temperature of the H_2_ pre-treatment.

The morphological analysis of MWCNT film was performed with a field-emission scanning electron microscope (SEM, Zeiss LEO 1530, Oberkochen, Germany) at an accelerating voltage of 10 kV. The MWCNTs are aligned by crowding effect and have a length of about 15 µm and an average diameter of about 20 nm (Figure 1b). Ni particles are visible at their top (bottom inset of Figure 1b), indicating a weak catalyst adhesion to Si_3_N_4_ and a dominant ‘‘tip growth’’ mechanism. 

A high-resolution transmission electron microscope (HRTEM, 200 CM Philips, Eindhoven, Netherlands) operating at 200 kV was used for the structural analysis the MWCNTs. The observations were performed on a piece of the film taken from the sample volume and deposited on a TEM copper grid. TEM analysis (Figure 1c) revealed the presence of MWCNTs with inner and outer diameters in the range of 5–15 nm and 10–35 nm, respectively. In addition, high-resolution transmission electron microscopy (HRTEM) measurements showed a typical interlayer spacing of 0.34 nm characteristic of the graphene in high oriented graphite.

We further checked the quality of the as-grown CNTs by Raman spectroscopy. The Raman scattering spectra were recorded at room temperature using a LabRam HR High-Resolution Raman Microscope HORIBA Jobin Yvon (Kyoto, Japan). We used the 632.8 nm (1.96 eV) excitation line from a He-Ne laser (spatial resolution of ~1 µm). Raman shift was calibrated by the Raman peak of crystalline silicon. We report in Figure 1d a Raman spectrum taken on the top of the film. The Raman spectrum presents two main peaks attributed to the D and G bands. The presence of defective graphitic structures accounts for the intense D-band and the broadening of the G-band at ~1600 ${\mathrm{cm}}^{-1}$. These bands result from the disordered layer at the top of the CNT film (right bottom inset of Figure 1b) [46]. 

The uniform MWCNT film forms an electrical connection between the two Pt-Ta pads, while the MWCNT film together with the Pt-Ta contacts isolated from the Si substrate by the Si_3_N_4_ layers, constitute a metal-insulator-semiconductor (MIS) heterostructure, which is initially in a high resistive state because of the thick Si_3_N_4_ barrier.

## 3. Results and Discussion

**Figure 2:**
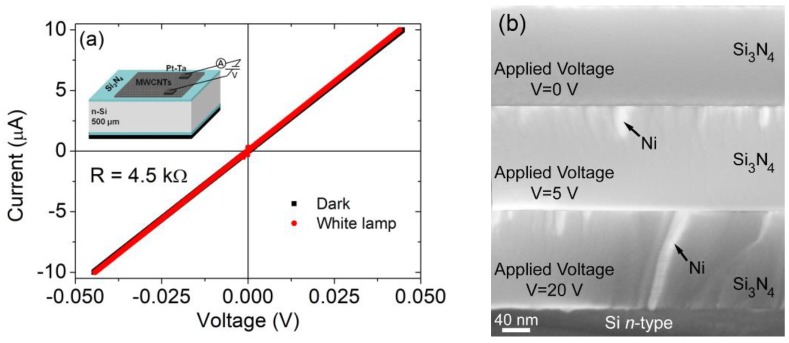
(**a**) I-V characteristics measured between the two top Ta-Pt pads for the Ta-Pt/CNT/Ta-Pt heterostructure. (**b**) SEM images showing the formation of Ni filaments in the Si_3_N_4_ layer after electric stress at increasing voltage.

Figure 2a shows the I-V characteristics measured between the two top Pt-Ta pads with the floating bottom contact. The I-V characteristics of the Pt-Ta/CNT/Pt-Ta structure are linear with a resistance of R≈5 kΩ, which does not change when the sample is exposed to the light from a 100 W incandescent lamp. The prevailing metallic character of the MWCNTs is the reason for the ohmic behavior and the poor photoconductivity.

The device was then subjected to a series of electric stresses at high voltage, which permanently damaged both the top and bottom Si_3_N_4_ layers, rendering them stable tunnel barriers. The damaging process is investigated in the SEM images of Figure 2b, related to the top Si_3_N_4_ layer, which show the gradual formation of conductive metallic filaments upon application of a growing voltage. For the top insulating layer, the metal of the filaments used for the CNT growth is mainly Ni, while Ta filaments from the Pt-Ta film are formed inside the bottom Si_3_N_4_ layer. Both metals, in contact with n-Si, can form Schottky junctions [47,48,49]. Due to the different work functions (5.04–5.35 eV for Ni and 4.0–4.8 for Ta) [50], the electron Schottky barrier is higher for Ni, typically around 0.7 eV [51], and lower for Ta, usually <0.5 eV [52].

Once the repeated electrical stress set the two Si_3_N_4_ barriers in a conductive and stable state, we started the systematic characterization of the MISIM device before and after the mechanical removal of the MWCNT film, that is, of the Pt-Ta_CNT/Si_3_N_4_/n-Si/Si_3_N_4_/Pt-Ta and the Pt-Ta/Si_3_N_4_/n-Si/Si_3_N_4_/Pt-Ta vertical structures. 

**Figure 3:**
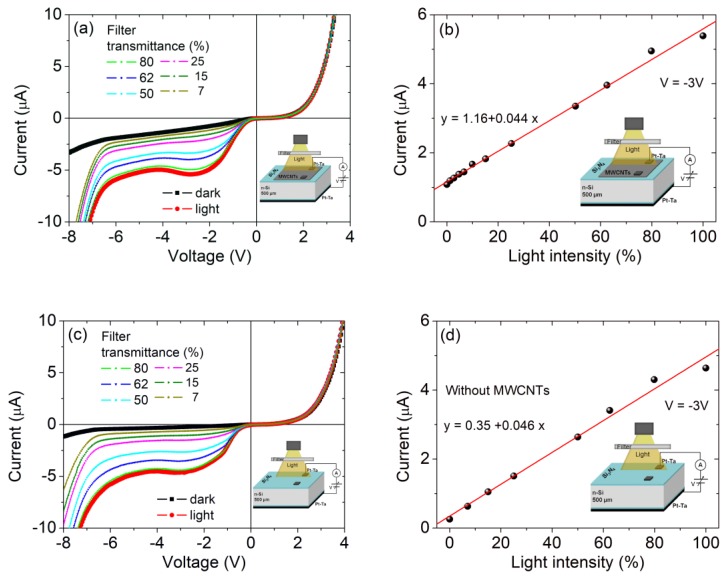
I-V characteristics and reverse current at –3 V as a function of the illumination intensity (percentage) of the Pt-Ta_CNT/Si_3_N_4_/n-Si/Si_3_N_4_/Pt-Ta (**a**,**b**) and Pt-Ta/Si_3_N_4_/n-Si/Si_3_N_4_/Pt-Ta (**c**,**d**) MISIM heterostructures.

Figure 3a shows the results of I-V measurements performed in the dark and under illumination by the incandescent lamp gradually attenuated by optical filters of different transmittance. 

The dark I-V characteristics show a rectifying behavior with the forward current for the positive bias on the top pad. The reverse current exhibits a dependence on the applied bias as well as a dramatic increase when the device is exposed to light. The light does not affect the forward current. We note a small peak on the reverse current at a bias around −2.9 V. At a lower voltage, for V < −6.5 V, the reverse current suddenly increases. However, we can exclude a breakdown that is expected at $V_{\mathrm{BD}}\approx$ −300 V, according to the semi-empirical formula of the one-sided step junction: $V_{\mathrm{BD}}\approx -\;60\;{\left(E_{G}/1.1\;\mathrm{eV}\right)}^{1.5}\;{\left(N_{D}/10^{16}{\;\mathrm{cm}}^{-3}\right)}^{-0.75}$ (here, $E_{G\;}\approx 1.1\;\mathrm{eV}$ is the semiconductor bandgap in eV, $N_{D}$ is the doping density in ${\mathrm{cm}}^{-3})$ [53].

Figure 3b demonstrates that the dependence of the reverse current at a given bias on the light intensity is monotonic. The linear response makes the device appealing for photodetection, with a differential responsivity of R=dI/dP≈1.7 mA/W (*I* is the current and P is the incident optical power with a maximum of $\approx 0.1{\;\mathrm{mW}\;\mathrm{cm}}^{-2}$).

To understand the role of the carbon nanotubes in the device, we mechanically removed the MWCNT film with a cotton swab and then performed the same optoelectric characterization on the Pt-Ta/Si_3_N_4_/n-Si/Si_3_N_4_/Pt-Ta MISIM heterostructure. Figure 3c,d show that the removal of the CNTs does not substantially change the properties of the device and demonstrate that the main effect of the MWCNT film is an increased reverse leakage current and photocurrent, as expected considering the wider contact surface area. 

We point out that the increased photocurrent is an important advantage of the device. Owing to its transparency, the MWCNT film increases the photosensitive area of the top contact to the whole surface of the film, while the reflecting Pt-Ta contact is photosensitive only in the region around its perimeter. 

**Figure 4:**
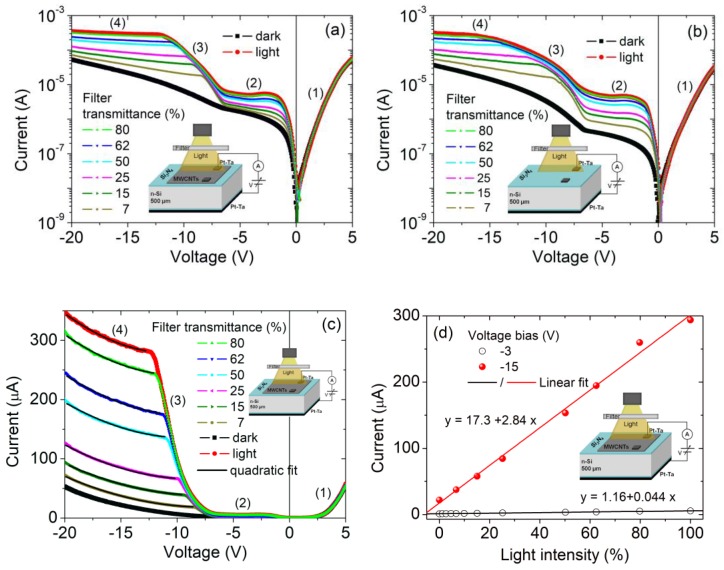
I-V characteristics of the Pt-Ta_CNT/Si_3_N_4_/n-Si/Si_3_N_4_/Pt-Ta (**a**) and Pt-Ta_CNT/Si_3_N_4_/n-Si/Si_3_N_4_/Pt-Ta. (**b**) Metal-insulator-semiconductor-insulator-metal (MISIM) heterostructures in the dark and under different degrees of illumination, on a semilogarithmic scale. (**c**) I-V characteristics on a linear scale. (**d**) Reverse current at −3 V and −15 V as a function of the illumination intensity (percentage) of the Pt-Ta_CNT/Si_3_N_4_/n-Si/Si_3_N_4_/Pt-Ta structure.

The similar behavior of the MISIM structures, with and without CNTs, is maintained when a wider voltage bias is applied. The I-V curves shown in Figure 4a,b on the semilogarithmic scale highlight an additional feature, that is, a sudden raise in the reverse current for V < −6.5 V, which leads to a second photocurrent plateau at a higher reverse bias. Figure 4c shows that for V < −10 V, the I-V curves corresponding to the second plateau are well fitted by a quadratic law $I\sim V^{2},$ indicating a space-charge limited conduction mechanism [54] that could arise from trapping in the two insulating barriers, the low doped Si as well as the accumulation of photogenerated holes at Si_3_N_4_/Si interface. The corresponding series resistance decreases from ∼300 kΩ in the dark to ∼150 kΩ at the highest light intensity.

More importantly, Figure 4d, displaying the photocurrent at a given bias versus light intensity, demonstrates that the linear behavior is kept and that a photocurrent gain occurs at high reverse bias (the gain is ~65 at V = −15 V). Such a gain enhances the photoresponsivity of the device to ∼110 mA/W under the incandescent light of $0.1{\;\mathrm{mWcm}}^{-2}$, a value competitive with that from other carbon-based photodetectors [32,37,55,56]. The bias-tunable photoresponsivity endows the device with additional functionalities and makes it suitable for lower or higher power applications.

**Figure 5:**
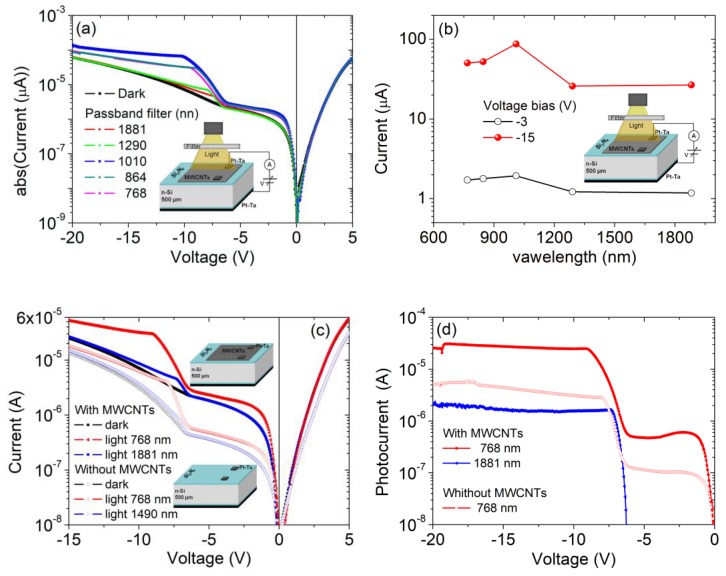
(**a**) I-V characteristics. (**b**) Photocurrent at V = −3 V and V = −15 V of the Pt-Ta_CNT/Si_3_N_4_/n-Si/Si_3_N_4_/Pt-Ta structure under the illumination of lights with different wavelengths. I-V characteristics (**c**) and photocurrent (current under illumination minus current in dark) (**d**) of the two vertical MISIM structures, with and without MWCNTs, for exposure to light of different wavelengths.

Figure 5 shows the I-V behavior of the vertical heterostructure, with and without MWCNT film, when illuminated from the top by lights of selected wavelengths, obtained from an incandescent lamp with passband optical filters of 50 nm bandwidth. Figure 5a,b demonstrate that the maximum reverse photocurrent is achieved for a wavelength close to the Si bandgap, i.e., at 1010 nm (corresponding to 1.01 eV), while wavelengths such as 1290 or 1881 nm below the Si energy bandgap have minor effects. This result confirms that photogeneration occurs mainly into the Si substrate, where a depletion layer is formed.

Indeed, the comparison of the photocurrent of the heterostructure before and after CNT removal, at the same wavelength (768 nm in Figure 5c,d), demonstrates that the sample with MWCNTs produces a higher photocurrent. We attribute this to the increased effective photosensitive junction area. While the reflective and opaque metal contact limits the photoconductive area to the perimeter region, the MWCNT film is transparent and increases the photosensitive surface area. Remarkably, Figure 5d shows that the MISIM with MWCNTs generates a photocurrent at 1881 nm (0.54 eV), which is not present when the CNTs are removed. The photocurrent at 1881 nm, being produced by light with energy below the Si bandgap, can be attributed to photoexcitation over the Schottky barrier by light absorbed in the MWCNT film. This observation indicates that the MWCNT film contributes to the photocharge generation as an active layer in addition to the function as antireflective and transparent conductive electrode, in agreement with previous work [32,33,34,36].

We finally note in Figure 5d that the photocurrent in the two plateau regions is weakly dependent on the reverse bias. 

### Band Model

**Figure 6:**
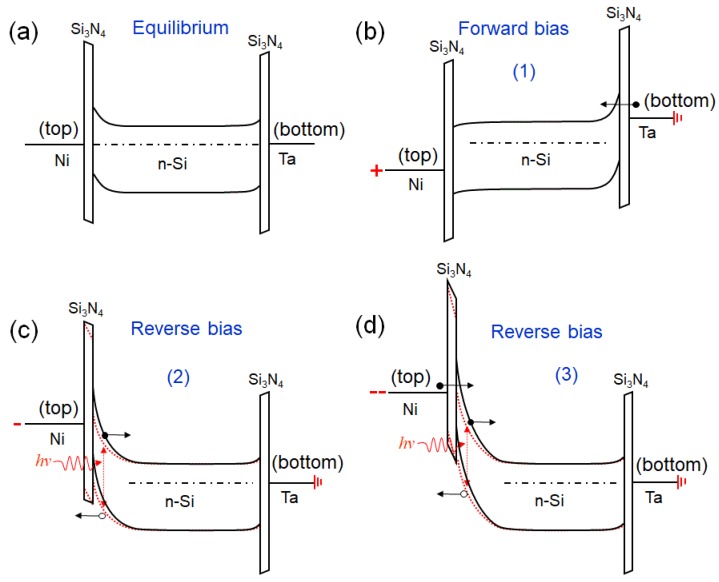
Band diagrams of the Pt-Ta_CNT/Si_3_N_4_/n-Si/Si_3_N_4_/Ta MISIM heterostructure in equilibrium: (**a**) forward, (**b**) lower, (**c**) higher, and (**d**) reverse bias. The red dotted curves represent the bands under illumination. Empty and full circles represent holes and electrons, respectively.

To explain the behavior of the device, we propose the band model shown in Figure 6, which accounts for the rectification and the high photoconductivity by considering the interplay between two asymmetric Schottky barriers [57]. We consider the electrically thinned Si_3_N_4_ layers as tunnel barriers, which can sustain a voltage drop but are almost transparent to the current. We assume that Ni forms a Schottky barrier at top slightly higher than the one formed by Ta at the bottom contact (Figure 6a).

In forward bias, when the top contact is positively biased, the voltage drop appears mostly across the highest resistance point that is the reverse-biased Schottky junction of the bottom contact (Figure 6b). Electrons are injected over this lower barrier, which can be further reduced by image force barrier lowering [57,58,59]. The vanishing depletion layer at the top contact and the rapidly growing current make the contribution of the photogenerated carriers negligible. 

In reverse bias (Figure 6c,d), the depletion layer at the top contact gradually extends to the maximum width, $W=\sqrt{2\varepsilon_{s}\left(V_{\mathrm{bi}}-V\right)/qN_{D}}\approx 5\;\mu m,$ at V = −20 V ($\varepsilon_{s}$ is the dielectric constant of Si, $V_{\mathrm{bi},}$, the built-in voltage q is the electron charge), causing the increase of the reverse current that is due to thermal or photogeneration. The hole current is enhanced when the applied voltage makes the Si_3_N_4_ valence band align with the Si valence band, which could explain the observed photocurrent peak around −2.9 V. For increasing bias, the band bending and the image force barrier lowering (not explicitly shown in the drawing) reduce the barrier width and height to a level that tunneling of electrons from the top metal to the underneath Si substrate takes place (Figure 6d) [57,58,59]. Such phenomena are responsible for the sudden increase of the current observed at V < −6.5 V in the dark. Under illumination, the accumulation of positive charge in the valence band at the Si_3_N_4_/Si top interface decreases the surface potential and increases the oxide voltage. The modified band diagrams shown by the red dotted lines in Figure 6c,d provide evidence of a reduced barrier resulting in enhanced tunneling [60]. This accounts for the steeper increase of the current under illumination observed for V < −6.5 V. In this voltage range, being $V\leq -6E_{G}/q$, this is the threshold used in the Si one-sided junction for the avalanche breakdown when impact ionization might also occur, contributing to the photocurrent gain. Finally, the plateaus observed at a higher reverse bias are caused by the series resistance [61,62], which changes with the illumination.

We note that a photocurrent with a similar double plateau has been reported in reverse biased Al/SiO_2_/n-Si MIS structures, where the second plateau has been attributed to the so called “soft pinning” of oxide voltage at large negative gate bias, i.e., limited variation of the oxide voltage, which restricts the further increase of the direct tunneling electron current [63]. Such an effect can be modeled as the quenching effect of a series resistance that depends on the illumination of the n-Si substrate.

From an application standpoint, we highlight that the two observed photocurrent regimes, below and above −6.5 V, add functionality to the device, which can operate at lower or higher voltages for the detection of weaker or stronger light intensity.

## 4. Conclusions

We have fabricated and studied Pt-Ta_CNT/Si_3_N_4_/n-Si/Si_3_N_4_/Pt-Ta vertical MISIM heterostructures and characterized their optoelectricbehavior. We have shown that such heterostructures exhibit rectifying behavior, which we have explained by a band model based on two asymmetric Schottky barriers. The forward current is due to electrons injected over the lower Schottky barrier, while the reverse current is characterized by two different conduction regimes attributed to thermal/photo generation and to the tunneling of majority carriers through the higher barrier, respectively. We have shown that the photocurrent increases linearly with the light intensity and is generated mainly in the Si substrate, although there might be a contribution from the MWCNT film. At high bias, the device achieves the responsivity of $110{\;\mathrm{mA}\;W}^{-1}$ under an incandescent light of $1{\;\mathrm{mW}\;\mathrm{cm}}^{-2}$ intensity, competitive with similar carbon-based devices.

This work demonstrates a promising device for photodetection and contributes to the understanding of the physics underlying the electrical behavior of CNT-based MIS heterojunctions needed for technological applications.
